# Propane-1,2-di­ammonium chromate(VI)

**DOI:** 10.1107/S1600536814002463

**Published:** 2014-02-08

**Authors:** Sonia Trabelsi, Manel Essid, Thierry Roisnel, Mohamed Rzaigui, Houda Marouani

**Affiliations:** a Laboratoire de Chimie des Matériaux, Faculté des Sciences de Bizerte, 7021 Zarzouna Bizerte, Tunisia; b Centre de Diffractométrie X, UMR 6226 CNRS, Unité Sciences Chimiques de Rennes, Université de Rennes I, 263 Avenue du Général Leclerc, 35042 Rennes, France

## Abstract

In the title mol­ecular salt, (C_3_H_12_N_2_)[CrO_4_], each chromate anion accepts six N—H⋯O and C—H⋯O hydrogen bonds from nearby propane-1,2-di­ammonium cations. Three of the four O atoms of the chromate anion accept these bonds; the remaining Cr—O bond length is notably shorter than the others. In the crystal, the anions and cations stack in layers lying parallel to (100): the hydrogen-bonding pattern leads to a three-dimensional network.

## Related literature   

For background to organic chromates, see: Chebbi & Driss (2002 ▶, 2004 ▶); Srinivasan *et al.* (2003 ▶). For the crystal structures of simple salts of the propane-1,2-di­ammonium cation, see: Pospieszna-Markiewicz *et al.* (2011 ▶); Gerrard & Weller (2002 ▶); Lee & Harrison (2003 ▶); Todd & Harrison (2005 ▶). For a discussion on hydrogen bonding, see: Brown (1976 ▶); Blessing (1986 ▶).
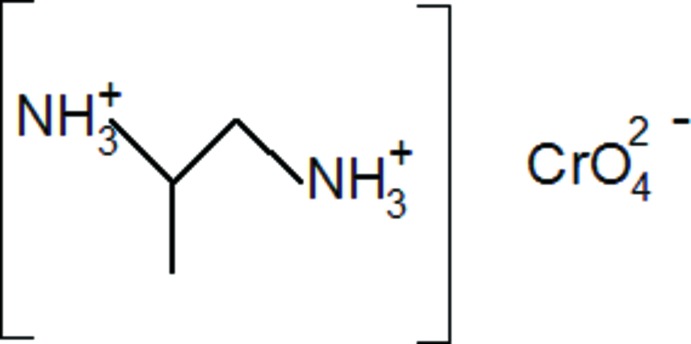


## Experimental   

### 

#### Crystal data  

(C_3_H_12_N_2_)[CrO_4_]
*M* *_r_* = 192.15
Monoclinic, 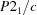

*a* = 5.6462 (2) Å
*b* = 15.8373 (5) Å
*c* = 8.4442 (3) Å
β = 106.779 (1)°
*V* = 722.94 (4) Å^3^
*Z* = 4
Mo *K*α radiation
μ = 1.54 mm^−1^
*T* = 150 K
0.55 × 0.44 × 0.31 mm

#### Data collection  

Bruker APEXII diffractometer
Absorption correction: multi-scan (*SADABS*; Sheldrick, 2002 ▶) *T*_min_ = 0.477, *T*_max_ = 0.620
6302 measured reflections
1659 independent reflections
1554 reflections with *I* > 2σ(*I*)
*R*_int_ = 0.032

#### Refinement  

*R*[*F* ^2^ > 2σ(*F* ^2^)] = 0.026
*wR*(*F* ^2^) = 0.071
*S* = 1.14
1659 reflections
110 parameters
H atoms treated by a mixture of independent and constrained refinement
Δρ_max_ = 0.34 e Å^−3^
Δρ_min_ = −0.52 e Å^−3^

### 

Data collection: *APEX2* (Bruker, 2006 ▶); cell refinement: *SAINT* (Bruker, 2006 ▶); data reduction: *SAINT*; program(s) used to solve structure: *SIR97* (Altomare *et al.*, 1999 ▶); program(s) used to refine structure: *SHELXL97* (Sheldrick, 2008 ▶); molecular graphics: *ORTEP-3 for Windows* (Farrugia, 2012 ▶) and *DIAMOND* (Brandenburg & Putz, 2005 ▶); software used to prepare material for publication: *WinGX* (Farrugia, 2012 ▶) and *CRYSCAL* (T. Roisnel, local program).

## Supplementary Material

Crystal structure: contains datablock(s) I. DOI: 10.1107/S1600536814002463/hb7193sup1.cif

Structure factors: contains datablock(s) I. DOI: 10.1107/S1600536814002463/hb7193Isup2.hkl

CCDC reference:

Additional supporting information:  crystallographic information; 3D view; checkCIF report

## Figures and Tables

**Table 1:** Selected bond lengths (Å)

Cr—O3	1.6182 (13)
Cr—O2	1.6378 (13)
Cr—O1	1.6711 (13)
Cr—O4	1.6879 (13)

**Table 2:** Hydrogen-bond geometry (Å, °)

*D*—H⋯*A*	*D*—H	H⋯*A*	*D*⋯*A*	*D*—H⋯*A*
N1—H1*A*⋯O4^i^	0.85 (3)	1.94 (3)	2.769 (2)	166 (2)
N1—H1*B*⋯O4^ii^	0.87 (3)	1.97 (3)	2.816 (2)	164 (2)
N1—H1*C*⋯O2^iii^	0.85 (3)	1.97 (3)	2.818 (2)	171 (2)
N2—H2*A*⋯O1^iv^	0.82 (3)	1.96 (3)	2.779 (2)	178 (2)
N2—H2*B*⋯O1^v^	0.85 (2)	1.91 (3)	2.748 (2)	173 (2)
N2—H2*C*⋯O4	0.85 (3)	1.95 (3)	2.795 (2)	171 (2)
C1—H1⋯O2^iv^	0.99	2.34	3.313 (2)	167
C3—H3*B*⋯O2^iii^	0.98	2.37	3.301 (2)	158

Symmetry codes: (i) 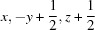; (ii) 
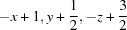
; (iii) 
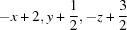
; (iv) 
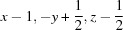
; (v) 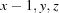.

**Fig. 1.**
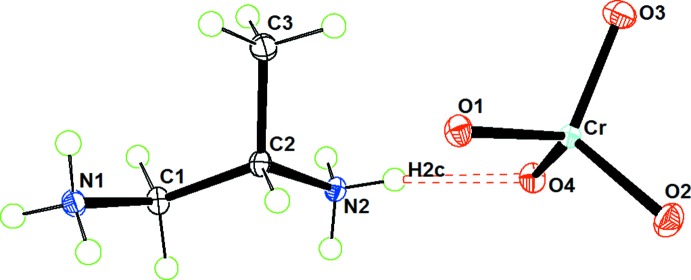
An ORTEP view of (I) with displacement ellipsoids drawn at the 30% probability level. H atoms are represented as small spheres of arbitrary radii. Hydrogen bonds are shown as dotted lines.

**Fig. 2.**
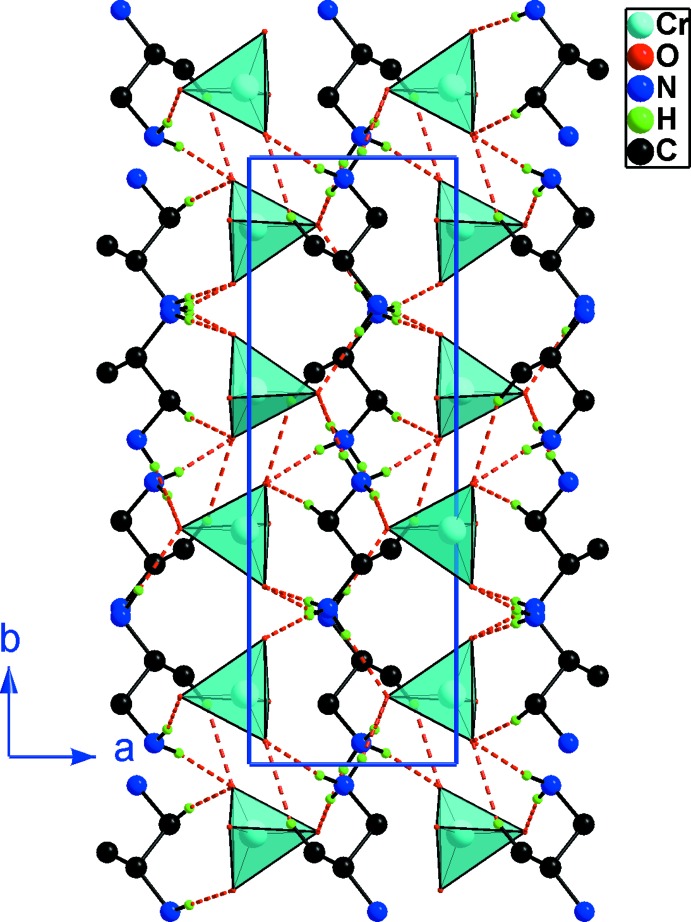
Projection of (I) along the c axis. The H-atoms not involved in H-bonding are omitted.

## supplementary crystallographic information

### 1. Comment

In this work, we report the preparation and the structural investigation of a new organic chromate, C_3_H_12_N_2_·CrO_4_ (I).

The asymmetric unit of (I) consists of one chromate anion and one propane-1,2-ammonium dication (Figure 1). The structure of the compound consists of discrete chromate ions stacked in layers parallel to the (100) plane, separated by organic cations (Figure 2). The structural cohesion is established by a three-dimensional network of N—H···O and C—H···O hydrogen bonds. Geometrical characteristics of the chromate anion are slightly different (Table 1). The distance Cr—O3 is notably the shortest (1.6182 (13) Å) because O3 is not applied in any hydrogen bond (Table 2) at the same time as Cr—O4 distance is the longest (1.6879 (13) Å) because O4 is applied in three hydrogen bonds. These geometrical features have also been noticed in other crystal structures (Chebbi & Driss, 2002; 2004; Srinivasan,*et al.*, 2003).

The 1,2-propanediammonium cation is characterized by N—C—C—N and N—C—C—C torsion angles of 164.88 (14) and -74.50 (19)°, respectively. Each organic entity is bounded to six different chromate anions through eight N—H···O and C—H···O hydrogen bonds forming a three dimensional network. Examination of the 1,2-propanediammonium cation shows that the bond distances and angles show no significant difference from those obtained in other simple salts involving the same organic groups (Pospieszna-Markiewicz,*et al.*, 2011; Gerrard,*et al.*, 2002; Lee, *et al.*, 2003; Todd,*et al.*, 2005).

The established weak H-bonds (Brown, 1976; Blessing, 1986) of types N—H···O and C—H···O involve oxygen atoms of the chromate anions as acceptors, and the protonated nitrogen atoms and carbon atoms of 1,2-diammoniumpropane as donors.

### 2. Experimental

CrO_3_ (0.10 g, 1 mmol) and 1,2-diaminopropane (0.13 ml, 1 mmol) were dissolved in distilled water (20 ml). The resulting solution was stirred for 30 min. and then evaporated slowly at room temperature. Yellow prisms of the title compound were obtained from the solution after one week.

### 3. Refinement

The hydrogen atoms bonded to N1 and N2 were located from a difference map and were allowed to refine. The rest of the H atoms were treated as riding, with C—H = 0.99 Å (methylene) or 0.98 Å (methyl) or 1.00 Å (methine), with *U*_iso_(H) = 1.2Ueq(parent C atoms) and 1.5Ueq(parent N or *C*-methyl atoms).

### Crystal data

**Table:**

(C_3_H_12_N_2_)[CrO_4_]	*F*(000) = 400
*M**_r_* = 192.15	*D*_x_ = 1.765 Mg m^−^^3^
Monoclinic, *P*2_1_/*c*	Mo *K*α radiation, λ = 0.71073 Å
Hall symbol: -P 2ybc	Cell parameters from 3695 reflections
*a* = 5.6462 (2) Å	θ = 2.8–27.5°
*b* = 15.8373 (5) Å	µ = 1.54 mm^−^^1^
*c* = 8.4442 (3) Å	*T* = 150 K
β = 106.779 (1)°	Prism, yellow
*V* = 722.94 (4) Å^3^	0.55 × 0.44 × 0.31 mm
*Z* = 4	

### Data collection

**Table:**

Bruker APEXII diffractometer	1659 independent reflections
Radiation source: fine-focus sealed tube	1554 reflections with *I* > 2σ(*I*)
Graphite monochromator	*R*_int_ = 0.032
CCD rotation images, thin slices scans	θ_max_ = 27.5°, θ_min_ = 3.6°
Absorption correction: multi-scan (*SADABS*; Sheldrick, 2002)	*h* = −7→6
*T*_min_ = 0.477, *T*_max_ = 0.620	*k* = −19→20
6302 measured reflections	*l* = −9→10

### Refinement

**Table:**

Refinement on *F*^2^	Primary atom site location: structure-invariant direct methods
Least-squares matrix: full	Secondary atom site location: difference Fourier map
*R*[*F*^2^ > 2σ(*F*^2^)] = 0.026	Hydrogen site location: inferred from neighbouring sites
*wR*(*F*^2^) = 0.071	H atoms treated by a mixture of independent and constrained refinement
*S* = 1.14	*w* = 1/[σ^2^(*F*_o_^2^) + (0.0303*P*)^2^ + 0.5214*P*] where *P* = (*F*_o_^2^ + 2*F*_c_^2^)/3
1659 reflections	(Δ/σ)_max_ = 0.001
110 parameters	Δρ_max_ = 0.34 e Å^−^^3^
0 restraints	Δρ_min_ = −0.52 e Å^−^^3^

### Special details

**Table:**

Geometry. All e.s.d.'s (except the e.s.d. in the dihedral angle between two l.s. planes) are estimated using the full covariance matrix. The cell e.s.d.'s are taken into account individually in the estimation of e.s.d.'s in distances, angles and torsion angles; correlations between e.s.d.'s in cell parameters are only used when they are defined by crystal symmetry. An approximate (isotropic) treatment of cell e.s.d.'s is used for estimating e.s.d.'s involving l.s. planes.
Refinement. Refinement of *F*^2^ against ALL reflections. The weighted *R*-factor *wR* and goodness of fit *S* are based on *F*^2^, conventional *R*-factors *R* are based on *F*, with *F* set to zero for negative *F*^2^. The threshold expression of *F*^2^ > σ(*F*^2^) is used only for calculating *R*-factors(gt) *etc*. and is not relevant to the choice of reflections for refinement. *R*-factors based on *F*^2^ are statistically about twice as large as those based on *F*, and *R*- factors based on ALL data will be even larger.

### Fractional atomic coordinates and isotropic or equivalent isotropic displacement parameters (Å^2^)

**Table:**

	*x*	*y*	*z*	*U*_iso_*/*U*_eq_	
Cr	0.97996 (5)	0.114804 (17)	0.67966 (3)	0.00751 (11)	
O1	1.0758 (2)	0.20744 (8)	0.77039 (15)	0.0120 (3)	
O2	1.0791 (3)	0.03771 (9)	0.81142 (16)	0.0154 (3)	
O3	1.0937 (3)	0.10356 (8)	0.52561 (16)	0.0146 (3)	
O4	0.6684 (2)	0.11150 (8)	0.60744 (16)	0.0124 (3)	
N1	0.5418 (3)	0.46519 (10)	0.7997 (2)	0.0112 (3)	
H1A	0.606 (4)	0.4428 (16)	0.894 (3)	0.017*	
H1B	0.456 (4)	0.5094 (16)	0.810 (3)	0.017*	
H1C	0.660 (5)	0.4816 (15)	0.763 (3)	0.017*	
N2	0.3804 (3)	0.25695 (10)	0.5858 (2)	0.0102 (3)	
H2A	0.289 (5)	0.2680 (15)	0.494 (3)	0.015*	
H2B	0.291 (4)	0.2454 (15)	0.648 (3)	0.015*	
H2C	0.467 (4)	0.2134 (16)	0.581 (3)	0.015*	
C1	0.3836 (3)	0.40086 (11)	0.6906 (2)	0.0107 (3)	
H1	0.2885	0.4275	0.5855	0.013*	
H2	0.2647	0.3774	0.7451	0.013*	
C2	0.5446 (3)	0.32984 (11)	0.6549 (2)	0.0097 (3)	
H3	0.6664	0.3121	0.7612	0.012*	
C3	0.6845 (4)	0.35472 (12)	0.5330 (2)	0.0141 (4)	
H3A	0.7805	0.3063	0.5133	0.021*	
H3B	0.7967	0.4016	0.5786	0.021*	
H3C	0.5667	0.3722	0.4284	0.021*	

### Atomic displacement parameters (Å^2^)

**Table:**

	*U*^11^	*U*^22^	*U*^33^	*U*^12^	*U*^13^	*U*^23^
Cr	0.00627 (16)	0.00701 (16)	0.00877 (16)	−0.00041 (10)	0.00140 (11)	−0.00009 (9)
O1	0.0121 (6)	0.0105 (6)	0.0129 (6)	−0.0018 (5)	0.0027 (5)	−0.0024 (5)
O2	0.0167 (7)	0.0130 (6)	0.0152 (6)	0.0031 (5)	0.0028 (5)	0.0037 (5)
O3	0.0151 (7)	0.0158 (6)	0.0145 (6)	−0.0032 (5)	0.0069 (5)	−0.0032 (5)
O4	0.0085 (6)	0.0122 (6)	0.0155 (6)	−0.0004 (5)	0.0021 (5)	−0.0011 (5)
N1	0.0127 (8)	0.0099 (7)	0.0110 (7)	0.0005 (6)	0.0035 (6)	−0.0014 (6)
N2	0.0105 (7)	0.0084 (7)	0.0116 (7)	−0.0006 (6)	0.0029 (6)	−0.0004 (6)
C1	0.0086 (8)	0.0102 (8)	0.0123 (8)	0.0002 (7)	0.0016 (7)	−0.0014 (6)
C2	0.0085 (8)	0.0085 (8)	0.0112 (8)	−0.0006 (6)	0.0014 (6)	−0.0011 (6)
C3	0.0135 (9)	0.0125 (8)	0.0185 (9)	−0.0021 (7)	0.0080 (7)	−0.0019 (7)

### Geometric parameters (Å, º)

**Table:**

Cr—O3	1.6182 (13)	N2—H2B	0.85 (2)
Cr—O2	1.6378 (13)	N2—H2C	0.85 (3)
Cr—O1	1.6711 (13)	C1—C2	1.530 (2)
Cr—O4	1.6879 (13)	C1—H1	0.9900
N1—C1	1.486 (2)	C1—H2	0.9900
N1—H1A	0.85 (3)	C2—C3	1.520 (2)
N1—H1B	0.87 (3)	C2—H3	1.0000
N1—H1C	0.85 (3)	C3—H3A	0.9800
N2—C2	1.490 (2)	C3—H3B	0.9800
N2—H2A	0.82 (3)	C3—H3C	0.9800
			
O3—Cr—O2	109.08 (7)	N1—C1—C2	109.95 (14)
O3—Cr—O1	108.31 (6)	N1—C1—H1	109.7
O2—Cr—O1	109.94 (7)	C2—C1—H1	109.7
O3—Cr—O4	108.67 (7)	N1—C1—H2	109.7
O2—Cr—O4	109.78 (7)	C2—C1—H2	109.7
O1—Cr—O4	111.01 (6)	H1—C1—H2	108.2
C1—N1—H1A	108.1 (16)	N2—C2—C3	108.76 (14)
C1—N1—H1B	111.1 (16)	N2—C2—C1	108.00 (14)
H1A—N1—H1B	110 (2)	C3—C2—C1	113.43 (15)
C1—N1—H1C	112.0 (16)	N2—C2—H3	108.9
H1A—N1—H1C	107 (2)	C3—C2—H3	108.9
H1B—N1—H1C	108 (2)	C1—C2—H3	108.9
C2—N2—H2A	111.0 (16)	C2—C3—H3A	109.5
C2—N2—H2B	110.0 (16)	C2—C3—H3B	109.5
H2A—N2—H2B	108 (2)	H3A—C3—H3B	109.5
C2—N2—H2C	110.2 (16)	C2—C3—H3C	109.5
H2A—N2—H2C	110 (2)	H3A—C3—H3C	109.5
H2B—N2—H2C	108 (2)	H3B—C3—H3C	109.5
			
N1—C1—C2—N2	164.88 (14)	N1—C1—C2—C3	−74.50 (19)

### Hydrogen-bond geometry (Å, º)

**Table:**

*D*—H···*A*	*D*—H	H···*A*	*D*···*A*	*D*—H···*A*
N1—H1*A*···O4^i^	0.85 (3)	1.94 (3)	2.769 (2)	166 (2)
N1—H1*B*···O4^ii^	0.87 (3)	1.97 (3)	2.816 (2)	164 (2)
N1—H1*C*···O2^iii^	0.85 (3)	1.97 (3)	2.818 (2)	171 (2)
N2—H2*A*···O1^iv^	0.82 (3)	1.96 (3)	2.779 (2)	178 (2)
N2—H2*B*···O1^v^	0.85 (2)	1.91 (3)	2.748 (2)	173 (2)
N2—H2*C*···O4	0.85 (3)	1.95 (3)	2.795 (2)	171 (2)
C1—H1···O2^iv^	0.99	2.34	3.313 (2)	167
C3—H3*B*···O2^iii^	0.98	2.37	3.301 (2)	158

Symmetry codes: (i) *x*, −*y*+1/2, *z*+1/2; (ii) −*x*+1, *y*+1/2, −*z*+3/2; (iii) −*x*+2, *y*+1/2, −*z*+3/2; (iv) *x*−1, −*y*+1/2, *z*−1/2; (v) *x*−1, *y*, *z*.
